# Alterations of transcriptome signatures in head trauma-related neurodegenerative disorders

**DOI:** 10.1038/s41598-020-65916-y

**Published:** 2020-06-01

**Authors:** Hyesun Cho, Seung Jae Hyeon, Jong-Yeon Shin, Victor E. Alvarez, Thor D. Stein, Junghee Lee, Neil W. Kowall, Ann C. McKee, Hoon Ryu, Jeong-Sun Seo

**Affiliations:** 1 Gong-Wu Genomic Medicine Institute, Seoul National University Bundang Hospital, Seongnam, 13605 Republic of Korea; 2 Department of Biomedical Sciences, Seoul National University Graduate School, Seoul, 03080 Republic of Korea; 3 Genomic Institute, Macrogen Inc., Seoul, 08511 Republic of Korea; 4 Center for Neuromedicine, Brain Science Institute, Korea Institute of Science and Technology, Seoul, 02792 Republic of Korea; 5 Boston Univeristy Chronic Traumatic Encephalopathy (CTE) Center, Boston University School of Medicine, Boston, MA 02118 USA; 6 VA Boston Healthcare System, Boston, MA 02130 USA; 7 BU Alzheimer’s Disease Center and Department of Neurology, Boston University School of Medicine, Boston, MA 02118 USA

**Keywords:** Gene expression, Long-term potentiation, Neurotransmitters

## Abstract

Chronic traumatic encephalopathy (CTE) is a neurodegenerative disease that is associated with repetitive traumatic brain injury (TBI). CTE is known to share similar neuropathological features with Alzheimer’s disease (AD), but little is known about the molecular properties in CTE. To better understand the neuropathological mechanism of TBI-related disorders, we conducted transcriptome sequencing analysis of CTE including AD and CTE with AD (CTE/AD) post-mortem human brain samples. Through weighted gene co-expression network analysis (WGCNA) and principal component analysis (PCA), we characterized common and unique transcriptome signatures among CTE, CTE/AD, and AD. Interestingly, synapse signaling-associated gene signatures (such as synaptotagmins) were commonly down-regulated in CTE, CTE/AD, and AD. Quantitative real-time PCR (qPCR) and Western blot analyses confirmed that the levels of synaptotagmin 1 (SYT1) were markedly decreased in CTE and AD compared to normal. In addition, calcium/calmodulin-dependent protein kinase II (CaMKII), protein kinase A (PKA), protein kinase C (PKC), and AMPA receptor genes that play a pivotal role in memory function, were down-regulated in head trauma-related disorders. On the other hand, up-regulation of cell adhesion molecules (CAMs) associated genes was only found in CTE. Our results indicate that dysregulation of synaptic transmission- and memory function-related genes are closely linked to the pathology of head injury-related disorder and AD. Alteration of CAMs-related genes may be specific pathological markers for the CTE pathology.

## Introduction

Chronic Traumatic Encephalopathy (CTE) is a degenerative brain disorder that is derived from repeated head trauma^1–3^. CTE is most often found in athletes who have a history of repetitive hits to the head while playing contact sports such as hockey, boxing, rugby, soccer, wrestling and others^4^. CTE symptoms, which include changes in cognition, mood, or behavior, start 8-10 years after the repeated, traumatic brain damage. Memory loss, impaired judgment, and executive function deficit are examples of cognitive symptoms that are featured in CTE patients. Mood changes typically involve depression, paranoia, apathy, loss of motivation, or suicidality. Behavioral changes are primarily seen as impulse control problems such as substance abuse, aggression, or violence^5^.

The clinical diagnosis of CTE is defined by post-mortem neuropathology through autopsy, immunohistochemical analysis, and tau PET imaging. The staging system for grading the pathological severity of CTE is divided into four stages^6^. Neuropathological hallmarks of CTE are composed of aggregates of hyperphosphorylated tau protein and neurofibrillary tangles (NFTs). In Stage I, discrete foci of hyperphosphorylated tau are localized around brain blood vessels that are mostly found in the lateral and frontal cortices. In Stage II, multiple foci of hyperphosphorylated tau are localized deep in the cerebral sulci. Neurofibrillary pathology spreads from the epicenter to the superficial layers of the adjacent cortex. In Stage III, hyperphosphorylated tau pathology spreads to the frontal, temporal, parietal and insular cortices. Brain weight loss occurs in the frontal and temporal lobes. In Stage IV, there is serious atrophy of the frontal, temporal, anterior thalami, and white matter tracts. Hyperphosphorylated tau pathology affects most regions of the cerebral cortex.

CTE and AD have both distinct and common features in clinical and neuropathological aspects. Deposition of hyperphosphorylated tau and presence of NFTs are common neuropathological features of CTE and AD^7^. The location of NFTs and presence of amyloid beta (Aβ) plaques are the differences between CTE and AD. In CTE, NFTs are non-uniform and are predominantly found in the superficial cortical layers. Also, CTE has a small amount of Aβ plaque deposits unlike AD. In AD, NFTs are more uniform and are mainly found in the third and fifth layers of the cerebral cortex^8^. AD has significant amounts of Aβ plaque deposition.

CTE is associated with other neurodegenerative disorders, including AD, motor neuron disease (MND), Parkinson’s disease (PD), Lewy body disease (LBD), frontotemporal lobar degeneration (FTLD), and multiple system atrophy (MSA). Previous studies have shown that, of the 142 cases with CTE, 37% had CTE with other neuropathology^9^. However, the relative contribution of other pathological substrates to clinical symptoms in CTE with other neuropathology is unknown.

Although the neuropathological features of head trauma-related disorders are well-documented, the definitive diagnosis of TBI-related diseases only relies on medical history, mental status testing, and brain imaging. In addition, exact gene regulatory mechanisms and molecular pathways are not fully understood. Accordingly, in the present study, we proposed to determine changes in molecular properties and to identify biological markers for TBI-related diseases through transcriptome analysis. We performed genome wide RNA sequencing analysis of post-mortem human brain tissues with CTE, CTE and AD, and AD. We found that deregulation of synaptic transmission- and memory function-related genes are closely associated with the pathology of head injury-related disorder and AD. We also discovered that altered expression of CAMs associated genes may play an important role in CTE pathology.

## Results

### Transcriptome analysis of CTE, CTE/AD, and AD

**Figure 1:**
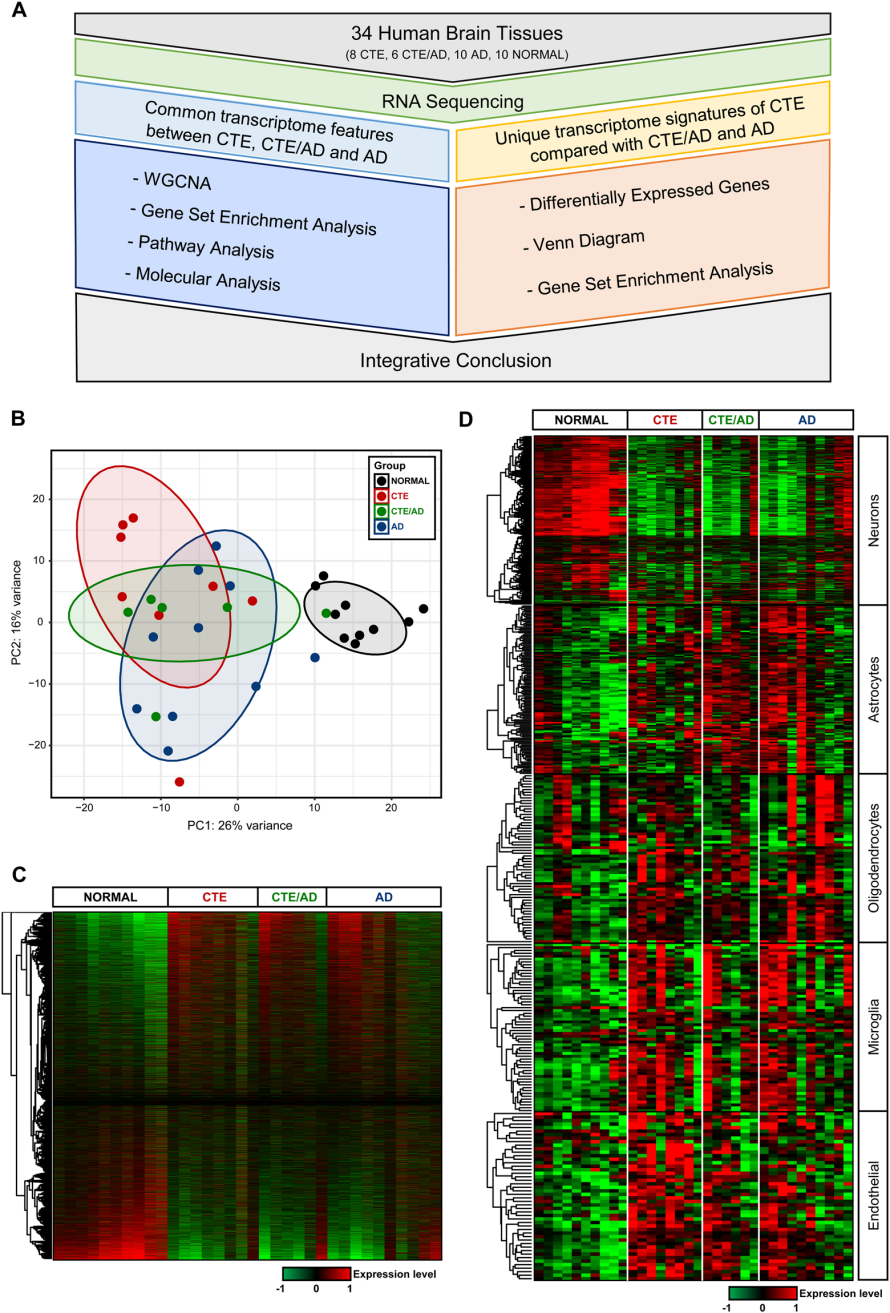
Global gene expression profiling. (**A**) Workflow of transcriptome analysis. (**B**) Principal component analysis (PCA) of gene expression profiles of all samples. CTE samples shown in red, CTE/AD samples shown in green, AD samples shown in blue, and normal samples shown in grey. PCA showed the separation of disease and normal groups. (**C**) Hierarchical clustering based on the RNA expression levels in each sample. CTE, CTE/AD, and AD showed a similar expression pattern in heatmap. (**D**) Expression levels of cell-type specific genes (543 Neurons, 120 Astrocytes, 61 Oligodendrocytes, 59 Microglia, and 48 Endothelial). 240 neuron-specific genes were mostly down-regulated in CTE, CTE/AD, and AD. 20 astrocyte-specific genes were up-regulated in CTE, CTE/AD, and AD. 6 oligodendrocyte-, 21 endothelial-, and 12 microglial-specific genes showed higher expressions in CTE, CTE/AD, and AD.

We collected 34 samples from the anterior temporal lobe of the human brain. The samples were taken from 8 individuals with CTE, 10 individuals with AD, 6 individuals with CTE/AD, and 10 normal individuals (Fig. 1A). For each sample, we recorded the diagnosis, gender, stage, and age of symptom onset (Supplementary Table S1). Among the 24 disease samples, most samples were verified as stage III or IV.

Through RNA sequencing, we obtained 9.24 Gb of RNA sequencing throughput on average and 84.17% of reads aligned to reference genome (Supplementary Table S2). To compute distances between samples, we conducted principal component analysis (PCA) based on the top 500 genes by variance across all samples. The expression pattern of CTE, CTE/AD, and AD was distinct from normal in the PC1 axis (26% variance) (Fig. 1B). Consistent with the PCA results, unsupervised hierarchical clustering analysis of 10,467 genes showed that there is a significant similarity of gene expression between CTE, CTE/AD, and AD (Fig. 1C and Supplementary Table S3).

Differentially expressed genes (DEGs) were determined based on q-value <0.05 and |Foldchange | ≥ 1.5 (Supplementary Table S4). We found 3,028 up-regulated and 2,713 down-regulated DEGs in CTE. 2,586 DEGs were up-regulated and 2,929 DEGs were down-regulated in CTE/AD. The number of up-regulated DEGs are 2,576 and down-regulated DEGs are 2,382 in AD. The FPKM levels were calculated to compare the expression levels of each disease (Supplementary Table S5).

The expression patterns of cell-type specific genes were similar to the results from PCA and hieriarchical clustering analysis (Fig. 1D). We obtained 543 neuron-, 120 astrocyte-, 61 oligodendrocyte-, 59 microglia-, and 48 endothelial- specific genes in CTE, CTE/AD, and AD (Supplementary Table S6). In neurons, 240 genes were commonly dysregulated in CTE, CTE/AD, and AD. We found that 20 astrocyte-, 6 oligodendrocyte-, 21 microglia-, and 12 endothelial-specific genes were commonly up-regulated in CTE, CTE/AD, and AD.

### Weighted gene co-expression network analysis of CTE, CTE/AD, and AD

**Figure 2:**
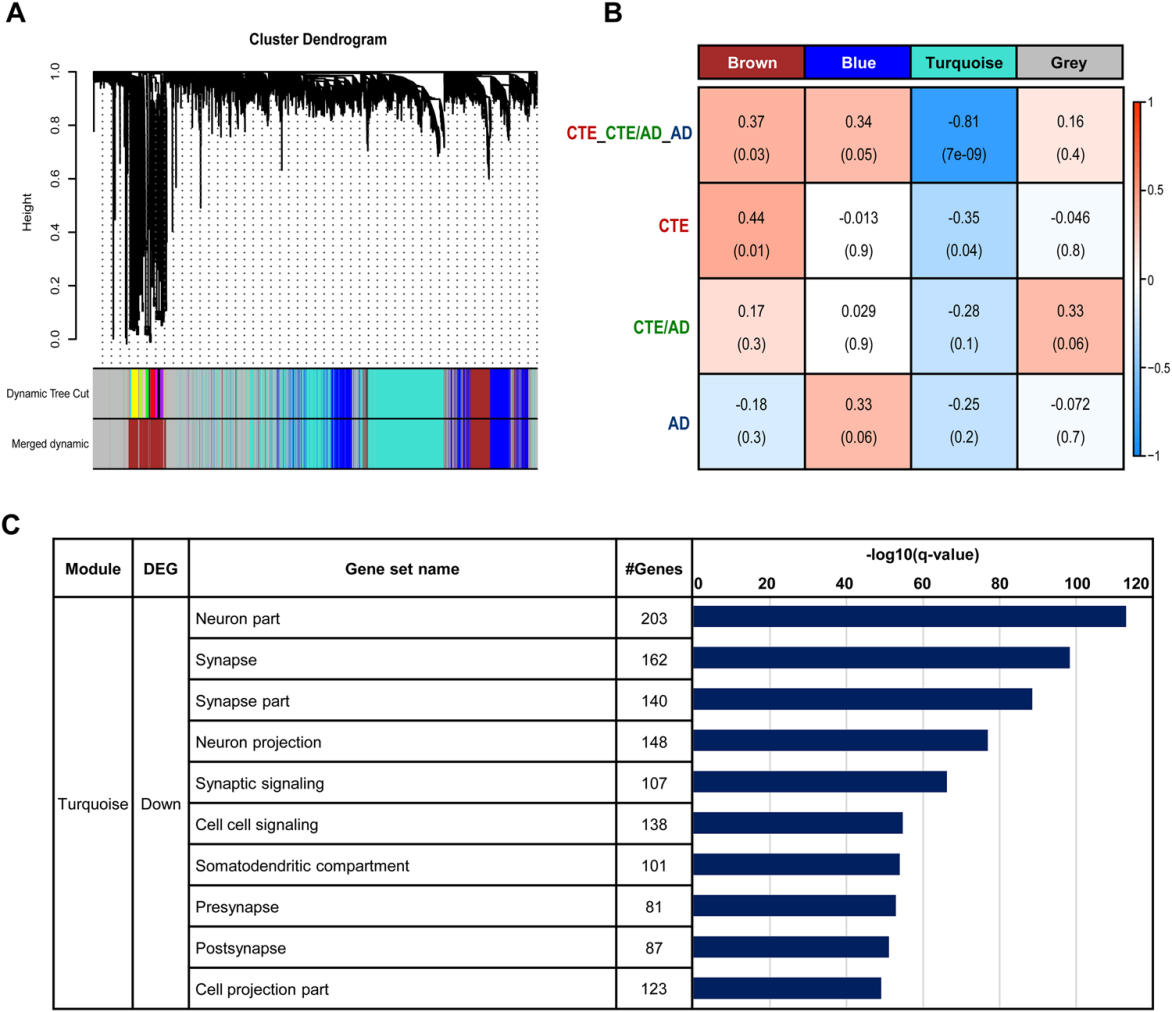
Turquoise module is significantly correlated with CTE, CTE/AD, and AD (**A**) Gene dendrogram obtained by clustering the dissimilarity based on topological overlap with the corresponding module colors indicated by the color row. Dynamic tree cut indicates an original color-coded module and merged dynamic represents merged module colors, which each contain a group of highly connected genes. A total of 4 modules were identified (turquoise, blue, brown, and grey). (**B**) Relationships of the detected modules and diseases. Each row in the table corresponds to the detected module, and each column corresponds to a disease. The Pearson’s correlation coefficient (PCC) values range from -1 (blue) to +1 (red) depending on the strength of the relationships. Each PCC value is accompanied by the corresponding p-value in brackets. Among the modules, the turquoise module was significantly correlated in CTE, CTE/AD, and AD. (**C**) Gene set enrichment analysis of DEGs in turquoise module. Among the gene sets of GO, neuron part, synapse, and synapse pathways were notably enriched in down-regulated genes.

To explore the neuropathological features of TBI-related human brain disorders, we performed weighted gene correlation network analysis (WGCNA). 10,467 genes between CTE, CTE/AD, and AD were used to construct gene co-expression networks. Based on the topological overlap of the genes, modules of co-expressed genes were identified by step-by-step network construction. The module labeled by colors was depicted in the hierarchical clustering dendrogram (Fig. 2A). We identified 4 modules, with a range in size from 1,716 genes in the blue module to 3,596 genes in the grey module. The grey module was excluded in the analysis because it was a collection of genes that could not be aggregated to other modules. We condensed the gene expression pattern within a module to a module eigengene, which is the first principal component of a module and is representative of the gene expression profiles of a module. To test whether or not module eigengenes are associated with diseases, we defined 3 traits: CTE, CTE/AD, and AD. Based on the PCA and hierarchical clustering analysis, we found a similar expression pattern between CTE, CTE/AD, and AD. As a result, we added a trait named CTE_CTE/AD_AD which is a trait including CTE, CTE/AD, and AD.

We obtained the relationships between the module eigengenes and the 4 traits (Fig. 2B). The results revealed that the brown module was positively correlated with the CTE_CTE/AD_AD trait. The turquoise module had the strongest negative correlation with CTE_CTE/AD_AD (R = −0.8, p < 2×10^−8^). We plotted a scatter plot of gene significance for CTE_CTE/AD_AD and module membership of genes in the turquoise module (Supplementary Fig. S1). We identified a number of genes of high significance for CTE_CTE/AD_AD as well as high module membership in the module. Through topological overlap matrix, we also discovered that the turquoise module was the most highly interconnected module (Supplementary Fig. S2). We represented gene symbol, locuslinkID, gene significance (GS) for CTE_CTE/AD_AD, module membership (MM), and p-values of all modules in Supplementary Table S7.

### Gene set enrichment analysis in CTE, CTE/AD, and AD

For modules with a strong negative correlation with the trait, the hub genes in the module should have negative GS and high positive MM. We defined the 1,603 hub genes of the turquoise module as GS < 0 and MM > 0.5 because turquoise module was negatively correlated with CTE_CTE/AD_AD trait. In case of the brown module, we identified 896 hub genes with positive GS and high positive MM (MM > 0.5) because brown module was positively correlated with CTE_CTE/AD_AD trait.

To identify the biological function associated with the modules, we performed GO enrichment analysis using up- and down-regulated genes in each module. In the turquoise module, 622 hub genes were commonly dysregulated in CTE, CTE/AD, and AD. Neuron part, synapse, and synapse part pathways were remarkably enriched in 622 down-regulated genes (Fig. 2C). Moreover, the number of genes belonging to the neuron pathway was the greatest in the turquoise module. In the brown module, 17 hub genes were commonly up-regulated in CTE, CTE/AD, and AD, and were not enriched in any pathways.

### Synaptic transmission-related genes were down-regulated in CTE, CTE/AD, and AD

**Figure 3:**
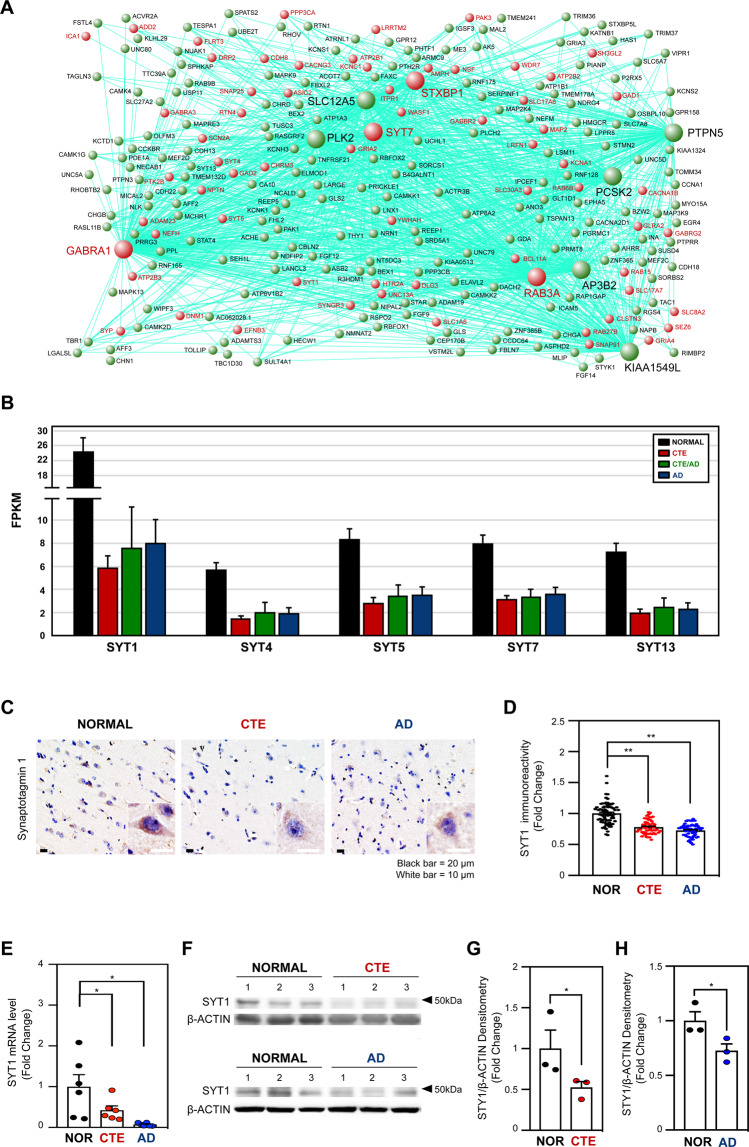
Synaptotagmin plays a key role in turquoise module (**A**) Network visualization of 266 genes in turquoise module. Each node indicates a gene. The genes with at least one connection when weighted cutoff value of >0.22 are shown. Among the 266 genes, 67 genes belonged to neuron part, synapse and synapse part pathways and were shown in red. The top 10 hub genes had higher connections with other genes and were represented with bigger nodes. (**B**) The FPKM level of synaptotagmin (*SYT1, SYT4, SYT5, SYT7*, and *SYT13*) in turquoise module. (**C**) The SYT1 immunoreactivity was markedly reduced in the cytosolic compartment of cortical neurons in CTE and AD postmortem brain compared to normal subject. The nuclei were counter stained with hematoxylin (blue). Scale bars: black, 20 µm; white, 10 µm. (**D**) Densitometry analysis showed that the intensity of SYT1 is significantly decreased in the cortical neurons in CTE and AD postmortem brain compared to normal postmortem brain. The Student’s t-test (unpaired) was performed for statistical analysis. **Significantly different at *p* < 0.001. (**E**) The mRNA level of *SYT1* was significantly reduced in CTE and AD patients compared to normal subjects. (**F**) Synaptotagmin 1 (SYT1) protein was down-regulated in the cortex of postmortem brain of CTE and AD patients compared to normal subjects. Western blot data represent three cases of normal subjects, CTE patients, and AD patients, respectively. (**G**) Densitometry analysis showed that SYT1 protein level was significantly reduced in the postmortem brain of CTE patients. (**H**) Densitometry analysis showed that SYT1 protein level was significantly reduced in the postmortem brain of AD patients. *Significantly different from the normal subject at *p* < 0.05.

To visualize gene-gene interactions, we obtained gene-gene connection scores from WGCNA. We selected 622 commonly down-regulated genes in CTE, CTE/AD, and AD. We filtered gene-gene connection scores as weight cutoff value> 0.22. We selected the top 10 hub genes that were highly connected to other genes in the turquoise module, including *GABRA1, PTPN5, RAB3A, KIAA1549L, SLC12A5, PLK2, SYT7, AP3B2, STXBP1*, and *PCSK2*. (Supplementary Table S8). We represented 266 genes that were connected with the top 10 hub genes (Fig. 3A). Among the 266 genes, 67 genes belonged to neuron part, synapse, and synapse part pathways and were shown in red. In particular, *SYT7* was involved in the top 10 hub genes and were significantly down-regulated in CTE, CTE/AD, and AD. Other synaptotagmin family genes (*SYT1, SYT4*, and *SYT5*) were also involved in neuron, synapse, and synapse part pathways. Synaptotagmin family genes (*SYT1, SYT4, SYT5, SYT7*, and *SYT13*) were remarkably down-regulated in CTE, CTE/AD, and AD (Fig. 3B).

To verify whether SYT1 immunoreactivity is altered in CTE and AD brains, we performed immunohistochemistry. SYT1 immunoreactivity was intensely found within the cytosolic compartment of neurons in normal subjects. However, the SYT1 immunoreactivity was markedly reduced in the neuronal cell body and dendrites of CTE and AD patients (Fig. 3C). Densitometry analysis exhibited that SYT1 immunoreactivity is significantly decreased in the neuronal cell body of CTE and AD patients compared to normal subjects (Fig. 3D). To validate our transcriptomic results, we performed quantitative real-time PCR (qPCR) analysis from the postmortem brain tissue of CTE, AD, and normal subjects (Supplementary Table S9). In concordance with the transcriptome data, we found that *SYT1* mRNA level was significantly decreased in both CTE and AD patients compared to normal subjects (Fig. 3E). Moreover, Western blot and densitometry analyses showed that the protein level of SYT1 was decreased in both CTE and AD patients compared to normal subjects (Fig. 3F–H and Supplementary Fig. S3).

### Memory function-related genes were down-regulated in CTE, CTE/AD, and AD

**Figure 4:**
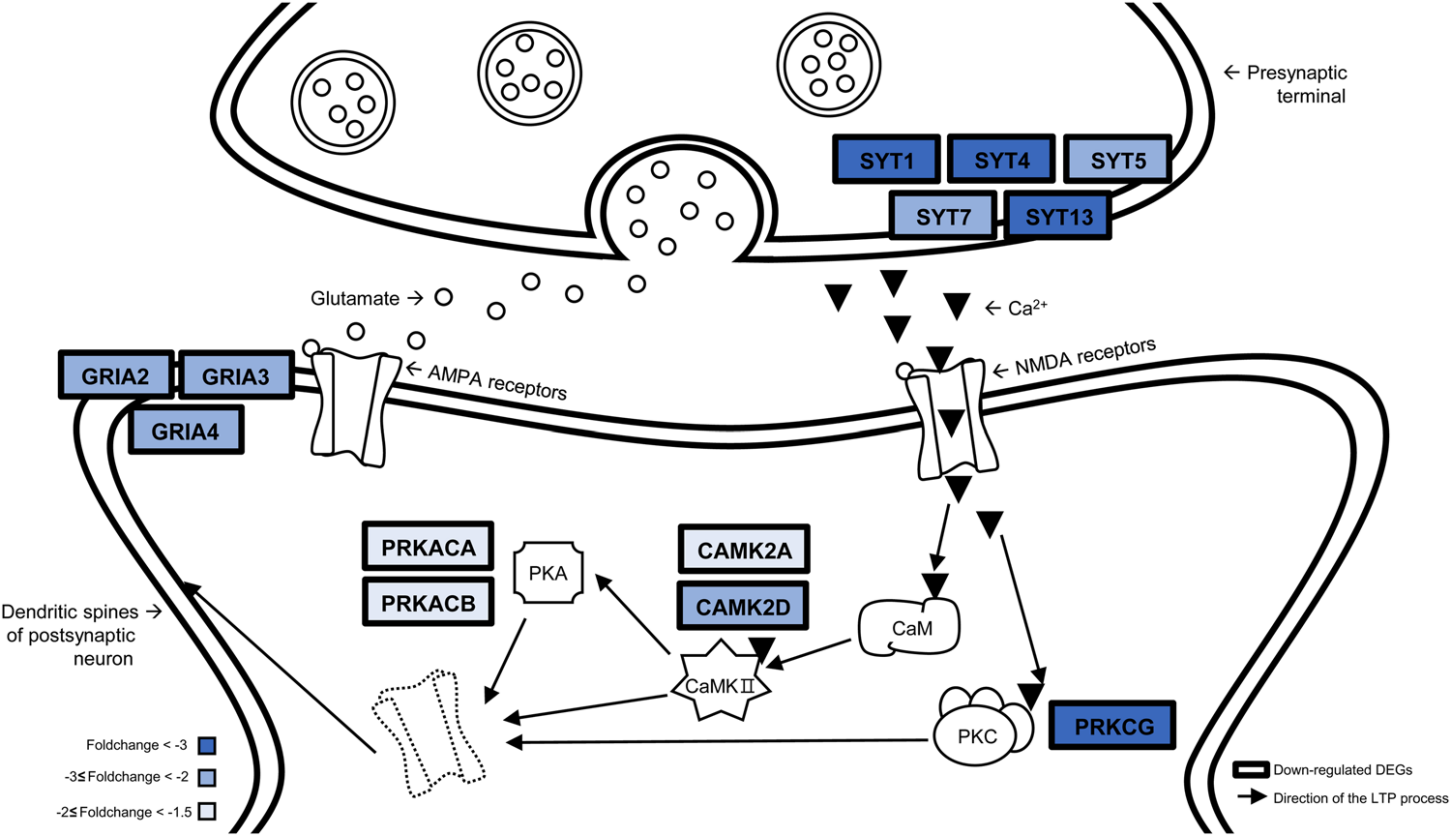
Long-term potentiation mechanisms involved in CTE, CTE/AD, and AD pathogenesis Memory function-related genes were significantly down-regulated in CTE, CTE/AD, and AD. The common DEGs of CTE, CTE/AD, and AD were shown in the scheme. The average foldchange levels of DEGs in CTE, CTE/AD, and AD were represented in blue. The arrows describe the long-term potentiation (LTP) process. (CaM = calmodulin, CAMKII = Ca2+/calmodulin-dependent protein kinase II, PKA = protein kinase A, PKC = protein kinase C).

Among the genes mentioned above, *SYT1* and *SYT7* were reported to play a critical role in memory function^10–12^. We also looked at the expression of other genes related to memory function. For example, the genes that play an important role in long term potentiation (LTP) process were prominently dysregulated in CTE, CTE/AD, and AD (Fig. 4). AMPA receptors contain four subunits, designated as *GluA1 (GRIA1), GluA2 (GRIA2), GluA3 (GRIA3)*, and *GluA4 (GRIA4)*. Among them, *GRIA2, GRIA3*, and *GRIA4* were strikingly down-regulated in all three disease groups (Supplementary Fig. S4A). CaMKII subfamily genes (*CAMK2A* and *CAMK2D*) were remarkably dysregulated in CTE, CTE/AD, and AD (Supplementary Fig. S4B). PKA catalytic subunits (*PRKACA* and *PRKACB*) were also down-regulated in CTE, CTE/AD, and AD (Supplementary Fig. S4C). *PRKCG*, one of the major forms of PKC, was commonly down-regulated in all three disease groups (Supplementary Fig. S4D).

### Cell adhesion molecules (CAMs) associated genes were up-regulated only in CTE

**Figure 5:**
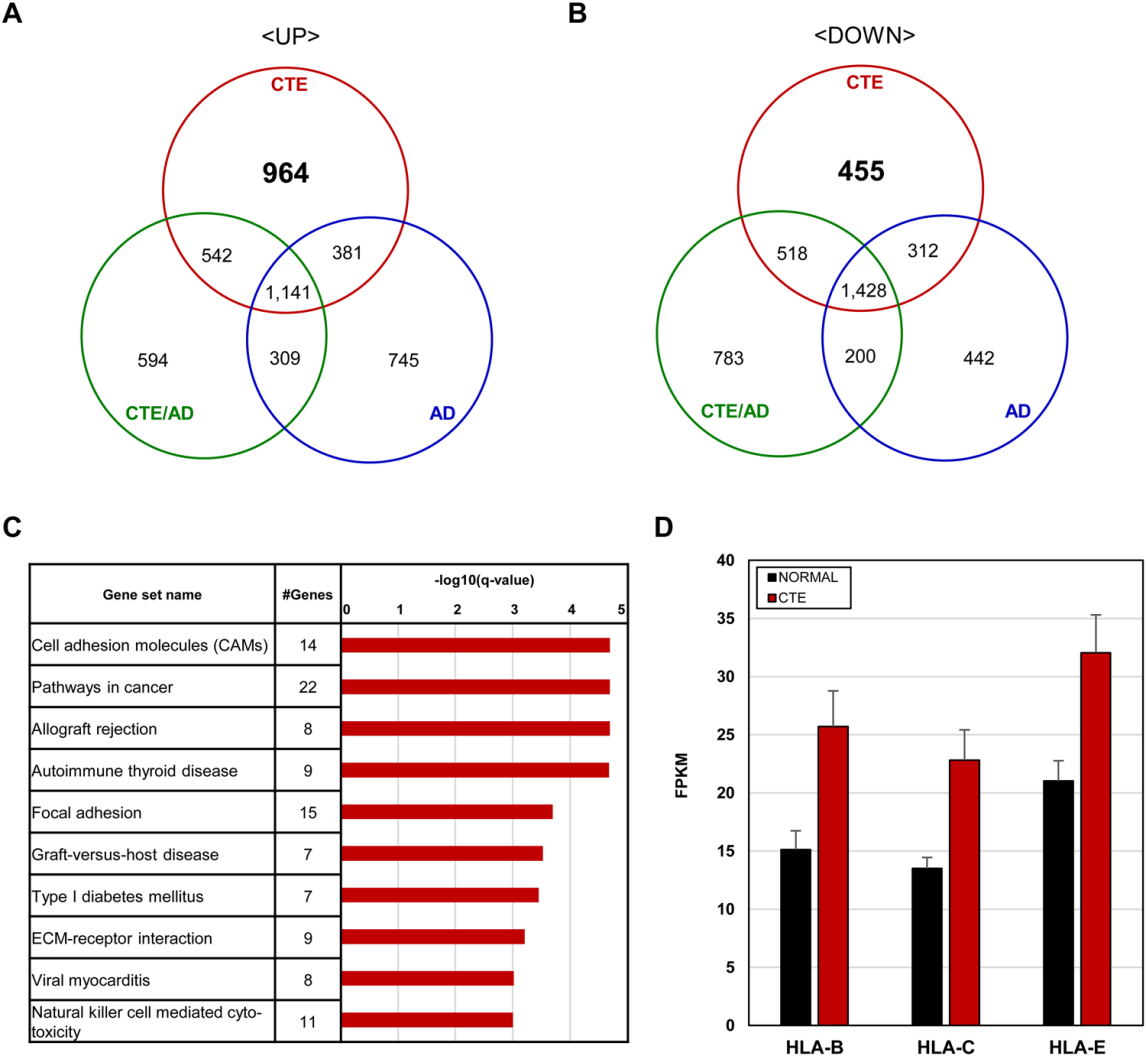
Unique transcriptome signatures of CTE. (**A**) Comparison of the number of up-regulated genes in CTE, CTE/AD, and AD. (**B**) Comparison of the number of down-regulated genes in CTE, CTE/AD, and AD. (**C**) Top 10 KEGG pathway analysis of up-regulated genes in CTE. Cell adhesion molecules (CAMs) pathway was highly enriched in CTE. (**D**) The FPKM levels of HLA genes (*HLA-B, HLA-C*, and *HLA-E)* in CTE.

Based on differentially expressed genes (DEGs) derived from each of the diseases, we identified the number of common and distinct DEGs in CTE, CTE/AD, and AD (Fig. 5A,B). Among them, we focused on 964 up-regulated and 455 down-regulated genes in CTE for investigating unique transcriptome signatures. In KEGG enrichment analysis of CTE, cell adhesion molecules (CAMs) pathway was shown to be highly enriched in up-regulated genes in CTE (Fig. 5C). Down-regulated genes were not enriched in any pathways. In CAMs pathway, MHC class I- related genes like *HLA-B, HLA-C*, and *HLA-E* were significantly up-regulated in CTE (Fig. 5D).

## Discussion

CTE is a progressive neurodegenerative disorder that leads to behavior, mood, and memory dysfunction. While the neuropathological features of CTE are demonstrated, the fundamental gene regulatory mechanisms and biological pathways of CTE-related diseases remain unclear. Previous our study revealed the mechanisms of how TBI causes neuropathological sequelae of tauopathy in CTE^13^. In this study, we focused on common and unique transcriptome features of CTE and compared them to those of CTE/AD and AD.

CTE, CTE/AD, and AD showed common gene expression changes by cell-type. Neuronal genes were down-regulated in CTE, CTE/AD, and AD. Neuron loss is known to be found not only in normal aging, but also in the early stage of disease development^14^. Neuron death correlates with the severity of memory impairments, and leads to an inability to relocate neuronal organization of cerebral structures and add new neurons to them. Therefore, dysregulation of neuron impairs normal memory functions and learning process in CTE, CTE/AD, and AD. Up-regulation of the genes of astrocytes, oligodendrocytes, endothelial cells and microglia was shown in CTE, CTE/AD, and AD. Atrophy of astrocytes causes loss of synaptic connectivity, imbalance of neurotransmitter homeostasis, and neuronal death^15^. Oligodendrocytes are necessary for nerve repair after injury by preventing their cell death and maintaining myelin restoration^16^. Microglia are recognized as essential players in maintaining brain homeostasis and protecting the brain from infections and insults^17,18^. Microglia also exert a neuroprotective role to phagocytose and clear Aβ aggregates in AD^19,20^. Endothelial cells play a pivotal role in maintaining cardiovascular homeostasis^21^. Based on these results, we assumed that changed gene expression of astrocytes, oligodendrocytes, microglia, and endothelial cells has a great impact on various biological mechanisms in CTE, CTE/AD, and AD.

Changes in expression of synapse- and synaptic transmission- related genes represented the common transcriptome features of CTE, CTE/AD, and AD. Synapses are essential for neuronal function and communication. They connect the neurons in the brain by passing an electrical or chemical signal from neuron to neuron. Herein, we found that synaptotagmin genes (*SYT1*, *SYT4, SYT5, SYT7* and *SYT13*) were significantly down-regulated in CTE, CTE/AD, and AD. Synaptotagmins are Ca^2+^-binding protein that play a pivotal role in vesicle fusion to the synaptic membrane. SYT1 and SYT7 function as the main Ca^2+^ sensors for fast and slow presynaptic vesicle exocytosis, respectively. Previous studies have shown that SYT1 and SYT7 act as redundant Ca^2+^ sensors for AMPA exocytosis during LTP^10,11^. Therefore, we assumed that down-regulation of synaptotagmins impacts memory function in CTE, CTE/AD, and AD. Previous studies have shown that synaptic dysfunction results in cognitive impairment in AD and other dementias^22,23^. Our result suggests that dysregulation of synaptic transmission correlates with cognitive deficits and memory dysfunction in CTE as well as AD.

In the current study, we found down-regulation of α (alpha) and δ (delta) chains of CaMKII (*CAMK2A* and *CAMK2D*) in CTE, CTE/AD, and AD. CaMKII is a major synaptic protein and important mediator in the LTP process^24^. LTP process is the persistent strengthening of synaptic transmission and this process underlies the molecular mechanism of learning and memory. Glutamate is released from the presynaptic terminal and binds to its specific receptors at the post synapse. Glutamate displaces Mg^2+^ from the NMDA receptors and Ca^2+^ flows through the opened NMDA receptors. CaMKII detects the uptake of Ca^2+^ and thus triggers the biochemical cascade that enhances synaptic transmission^25^. CaMKII alters glutamate susceptibility by phosphorylating the AMPA receptor^26–29^. It has been reported that knock out mice of CAMK2A reduce the LTP process by half^30^.

*PRKACA* and *PRKACB*, which encode the catalytic subunits of PKA, were down-regulated in CTE, CTE/AD, and AD. PKA (protein kinase A), a cyclic AMP (cAMP)-dependent protein kinase, played a pivotal role in the LTP process^31^. PKA phosphorylates the GluA4 and GluA1 subunits to regulate the synaptic incorporation of AMPA receptors. *PRKCG*, one of the isozymes of the PKC, was significantly down-regulated in CTE, CTE/AD, and AD. PKC is a family of serine/threonine protein kinases and is involved in neuronal functions, such as modulation of ion channel and synaptic transmission^32–34^. PKC decreases Mg^2+^ affinity in the NMDA receptor channel to increase channel open time, which enhances the response of NMDA receptors^35,36^. PKC phosphorylates the GluA1 and GluA4 subunits of AMPA receptors to alter glutamate sensitivity^37^. Especially, PRKCG modulates the GluA4 subunit of AMPA receptors by directly binding to GluA4^38^. Previous studies demonstrated that *PKCγ (PRKCG)* mutant mice showed impaired LTP process^39,40^.

On the other hand, dysregulation of *GRIA2*, *GRIA3*, and *GRIA4* gene expression was also found in CTE, CTE/AD, and AD. AMPA receptors are consisted of 4 subunits (GRIA1-4). AMPA receptors regulate most of the excitatory synaptic transmission^41^. AMPA receptors are known to be phosphorylated by protein kinases such as PKA, PKC, and CaMKII. Phosphorylation of AMPA receptors potentiates their function. Among the AMPA receptors, GRIA1 (GluA1) and GRIA4 (GluA4) mainly act on the LTP process^42^.

Notably, the unique transcriptome signature of CTE was associated with CAMs (cell adhesion molecules). CAMs mediate interactions between cells and the surrounding extracellular matrix that are essential for the process of controlling cell survival, activation, migration, and proliferation^43^. In the brain, CAMs are important for neural network formation such as axon-axon contacts, axon-astrocyte contacts, synapse formation, and regulation of synaptic structure^44,45^. In addition, MHC class I molecules, which are expressed in neurons, showed remarkable expression changes in CTE. MHC class I molecules regulate neuronal differentiation and affect synaptic plasticity, axonal regeneration, and T cell-mediated response^46–48^. Our data implies that MHC class I molecules may contribute to the pathogenesis of CTE, but the exact mechanism remains to be investigated in future studies.

**Figure 6:**
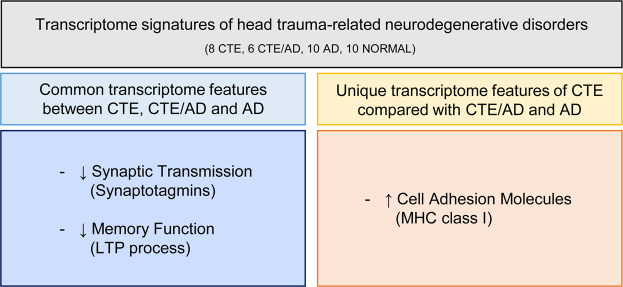
A schematic table of findings.

In summary, we identified alterations of common and unique transcriptome signatures in head trauma-related diseases (Fig. 6). Deregulation of synaptic transmission and memory function-associated transcriptomes were commonly affected in CTE, CTE/AD, and AD. On the other hand, up-regulation of CAMs-associated transcriptome signatures was found to be unique in CTE. Thus, altered transcriptome signatures provide insight on the understanding of molecular mechanisms of head trauma-related disorders, and these signatures may be new biological markers of CTE.

## Materials and Methods

### Human tissues

Neuropathological processing of 10 control, 8 CTE, 6 CTE/AD, and 10 AD human brain samples was performed using procedures previously established by the Boston University Alzheimer’s Disease Center (BUADC) [20]. Next of kin provided informed consent for participation and brain donation. Institutional review board approval for ethical permission was obtained through the BUADC and CTE center. This study was reviewed by the Institutional Review Board of the Boston University School of Medicine (Protocol H-28974) and was approved for exemption because it only included tissues collected from post-mortem subjects not classified as human subjects. The study was performed in accordance with institutional regulatory guidelines and principles of human subject protection in the Declaration of Helsinki. Clinical features including gender, stage, age of symptom onset, and regional pathology were described in Supplementary Table S1.

CTE is characterized pathologically by frontal and temporal lobe atrophy. Especially, the temporal lobe, including the hippocampus and the surrounding hippocampus regions, is critical for memory function. The temporal lobe is also involved in the primary organization of sensory input. The most common symptoms of neurodegenerative diseases are memory problems and sensory processing disorder. Moreover, hyperphosphorylated tau pathology is found in most regions of the cerebral cortex and the temporal lobe in CTE. Thus, temporal lobe dysfunction is highly associated with neurodegenerative processes and the neuropathology in CTE. In this context, we selected the temporal lobe of post-mortem brain for the transcriptome analysis.

### Transcriptome sequencing and analysis

Total RNA was extracted using the Illumina TruSeq RNA sample preparation kit and was sequenced on the Illumina HiSeq2000 platform. Raw reads were aligned to the human genome (GRCh37.p13) according to the STAR 2-pass method. STAR is an accurate alignment of high-throughput RNA-seq data. STAR is a two-step process that generates genome indexes files and maps the reads to the genome^49^. Duplicated reads were removed by Picard Markduplicate and filtered reads were further processed for variant calling using the GATK, including insertion/deletion realignment, base quality score recalibration, and haplotypecaller. According to Ensembl gene set, we used HTSeq to count the reads aligned to each gene^50^.

### Cell-type specific gene analysis

Cell-type specific genes (neurons, astrocytes, oligodendrocytes, endothelial, and microglia) were computed by making use of a large-scale human brain single-cell RNA seq dataset^51^. Cell-type specific genes were filtered by several criteria^52^. Genes with less than 50 reads across all samples were discarded. The remaining gene count data were analyzed by a Bayesian negative binomial regression model. Using numerical samples obtained by Markov chain Monte Carlo (MCMC), posterior probability was calculated and gene expression was enriched in one cell type compared to basal expression given by the regression. It was considered cell type-specific if the gene met two criteria: (1) abundant with 99.9% posterior probability in one cell and not in another, and (2) its expression in the enriched cells on average was fivefold greater than basal expression in numerical samples. We used filtered cell-type specific genes (1032 neurons, 191 astrocytes, 111 oligodendrocytes, 76 endothelial, and 118 microglia) and applied our gene expression data.

### Weighted Gene Co-expression network analysis (WGCNA)

The normalized read counts were used to construct signed co-expression networks using the WGCNA package in R. We used step-by-step network construction and the module detection method because auto construction method is not appropriate for our large data sets (10,467 genes). The network was constructed by obtaining a dissimilarity matrix based on the topological overlap. The adjacency matrix was calculated by raising the correlation matrix to a soft thresholding power of 14, which was chosen to attain scale-free topology. Gene dendrogram was generated and module colors were assigned. We calculated the module eigengene (ME) value, which was defined as the first principal component of a given module. Dendrogram cut height for module merging was 0.8. Merged module eigengenes were used to test the association of modules with diseases. Module membership (MM) was calculated as the correlation between gene expression levels and the module eigengene. Gene significance (GS) was also calculated as the correlation between gene and external traits. We defined hub genes using MM and GS values. If the module was positively correlated with the trait, we selected hub genes with positive GS and high positive MM (MM > 0.5). If the module was negatively correlated with the trait, we defined hub genes with negative GS and high positive MM (MM > 0.5). To facilitate biological interpretation, we applied DEGs of hub genes to the Molecular Signatures Database (MSigDB) of Gene Set Enrichment Analysis (GSEA)^53^. Gene Ontology (GO) gene set of MSigDB was selected to be analyzed. For network analysis, we used the WGCNA algorithm to calculate gene-gene interaction level. Based on gene-gene interaction level, the top 10 hub genes were visualized with VisANT (weight cut off >0.22).

### Differential gene expression analysis

For the gene expression profiling, we normalized read counts by using regularized log transformation method of DESeq. 2^54^. The calculated p-values were adjusted to q-values for multiple testing using the Benjamini-Hochberg correction. Genes with a |Foldchange | ≥ 1.5 and q-value < 0.05 were classified as significantly differentially regulated. For visualization, the PCA was plotted by disease using the plotPCA function in DESeq2. The normalized read counts were also used for hierarchical clustering analysis. Heatmaps were constructed using the dnet R package. FPKM (fragments per kilo base of exon per million mapped reads) for each gene was calculated and used for analyses. To find any gene sets significantly enriched in DEGs of CTE, we applied them to the Molecular Signatures Database (MSigDB) of Gene Set Enrichment Analysis (GSEA)^53^. Kyoto Encyclopedia of Genes and Genomes (KEGG) gene set of MSigDB was selected to be analyzed.

### Immunohistochemistry analysis

To detect SYT1 in human postmortem brain tissues, we performed immunohistochemistry as described previously^55^. Coronal plane of paraffin-embedded tissue sections (10 μm) were incubated with blocking solution after 3% H_2_O_2_ reaction for 1 hr. The tissue sections were incubated with anti-synaptotagmin 1 antibody (1:100 dilution; Abcam, ab131551) for 24 hr. After secondary antibody reaction, the tissue slides were further processed with Vector ABC Kit (Vector Lab PK-6100). DAB chromogen (Sigma D5637) was used to develop the immunoreactive signals. The nuclei were counterstained with hematoxylin. The tissue slides were examined under a bright field microscope and the intensity of immunoreactivity was analyzed using Multi-Gauge Software (Fuji photo film Co, Ltd. Japan).

### Western blot analysis

Western blot analysis was performed as previously described^13,56^. For the detection of SYT1 proteins, the blots were probed with anti-synaptotagmin 1 (1:100 dilution; Abcam, ab131551) and anti-β-actin (1:10000; Sigma Aldrich, St Louis, MO, USA) antibodies, followed by treatment with the appropriate secondary antibodies conjugated to horseradish peroxidase (Pierce, 170-6515 and 170-6516). Immunoreactivity was detected using an enhanced chemiluminescence (ECL) kit (Thermo Scientific, Waltham, MA, USA).

### Quantitative real-time PCR

Total RNA was extracted from the frozen brain tissues using TRIzol reagent (MRC, TR118) as previously described^13,56^. Fifty nanograms of RNA was used as a template for quantitative RT-PCR amplification, using SYBR Green Real-time PCR Master Mix (Toyobo, QPK-201, Osaka, Japan). Primers were standardized in the linear range of the cycle before the onset of the plateau. The primer sequences are shown in Supplementary Table S9. GAPDH was used as an internal control. Real-time data acquisition was performed using a LightCyler96 Real-Time PCR System (Roche Diagnostics, Indianapolis, IN, USA) under the following cycling conditions: 95 °C for 1 min× 1 cycle, and 95 °C for 15 s, followed by 60 °C for 1 min × 45 cycles. The relative gene expression was analyzed using the LightCyler96 software and expressed as Ct, the number of cycles needed to generate a fluorescent signal above a predefined threshold.

## Supplementary information

Supplementary Figure 1-4.

Supplementary Table 1.

Supplementary Table 2.

Supplementary Table 3.

Supplementary Table 4.

Supplementary Table 5.

Supplementary Table 6.

Supplementary Table 7.

Supplementary Table 8.

Supplementary Table 9.

## Data Availability

The RNA sequencing data are available under the European Nucleotide Archive (ENA) accessions no. ERP110728.
