# Supplementary material for: Alterations of transcriptome signatures in head trauma-related neurodegenerative disorders
Source: Sci Rep. 2020 Jun 1;10:8811. doi: 10.1038/s41598-020-65916-y (PMC7264177; doi:10.1038/s41598-020-65916-y)
Supplement: Supplementary file 1 — Supplementary Figure 1-4. [file 41598_2020_65916_MOESM1_ESM.pdf]

Supplementary Information

Alterations of transcriptome signatures in head trauma-related neurodegenerative disorders

Hyesun Cho, Seung Jae Hyeon, Jong-Yeon Shin, Victor E Alvarez, Thor D Stein, Junghee Lee, Neil W Kowall, Ann C McKee, Hoon Ryu, Jeong-Sun Seo

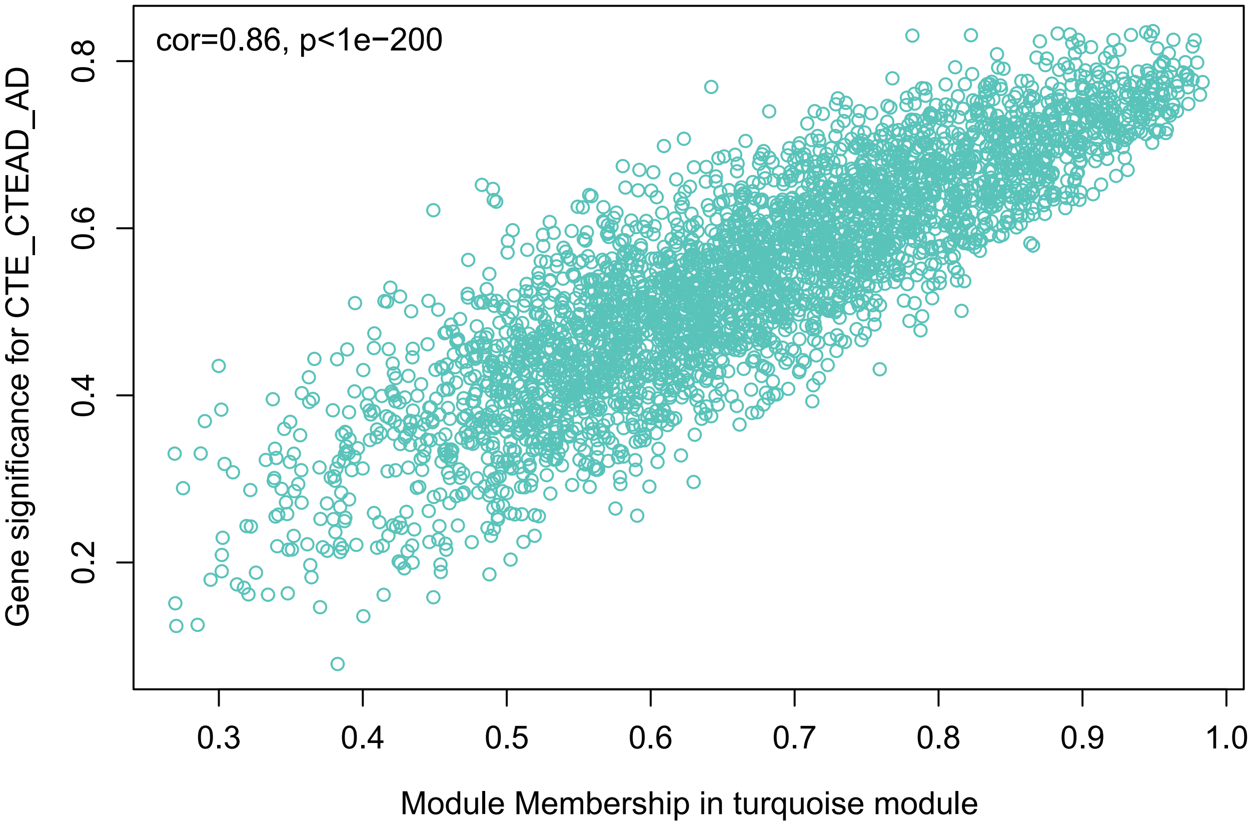

Supplementary Fig. S1. The correlation between gene significance for CTE_CTE/AD_AD and module membership in turquoise module. Gene significance for CTE_CTE/AD_AD status and module membership in turquoise module was significantly correlated.

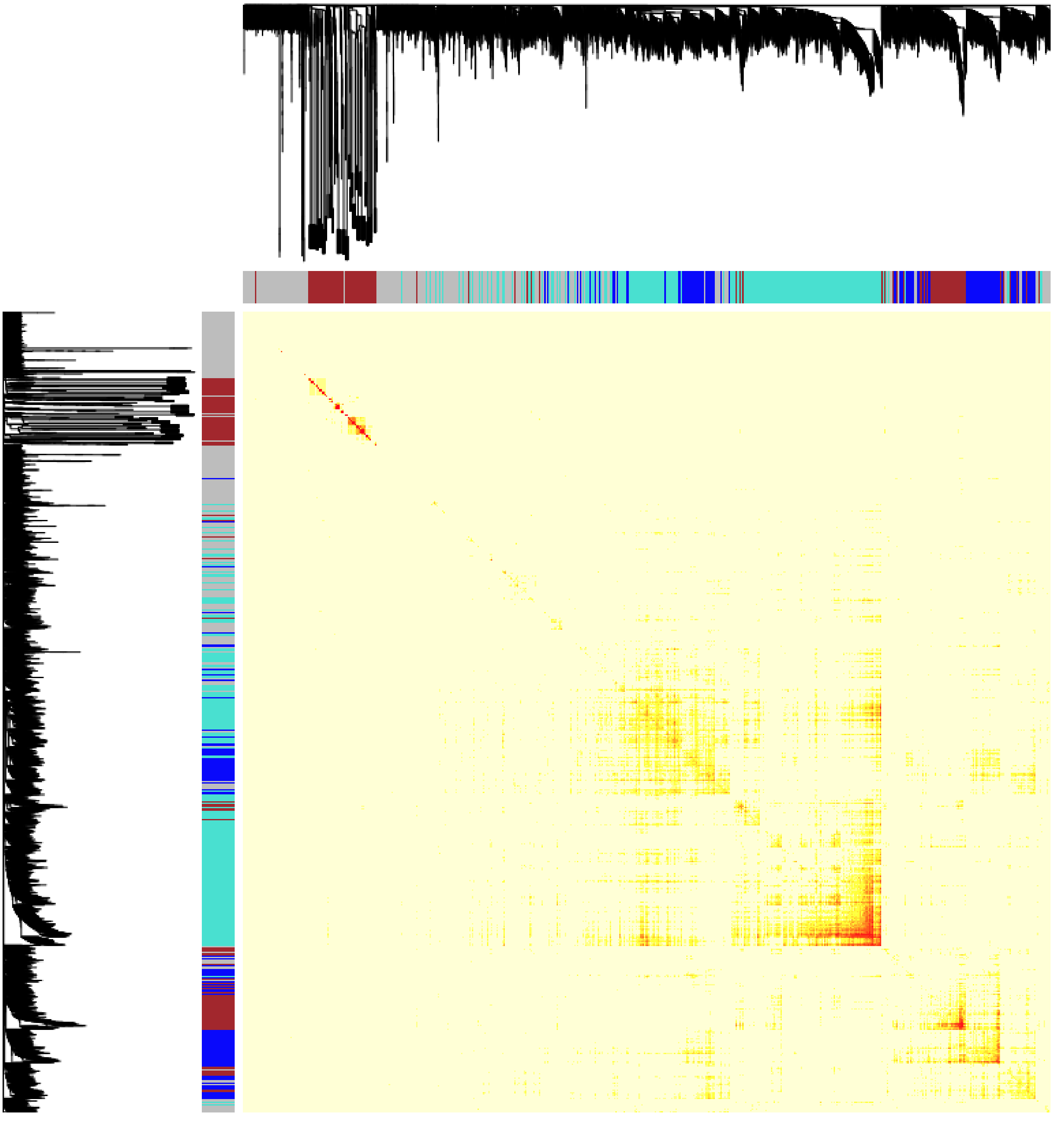

Supplementary Fig. S2. Topological overlap matrix plot.
The heatmap illustrates the topological overlap matrix (TOM) among all genes in 34 samples (8 CTE, 6 CTE/AD, 10 AD and 10 normal). Each row/column corresponds to genes. Left side and top indicate hierarchical clustering dendrogram and modules. Light colors represent lower topological overlap and a darker colors indicate higher topological overlap.

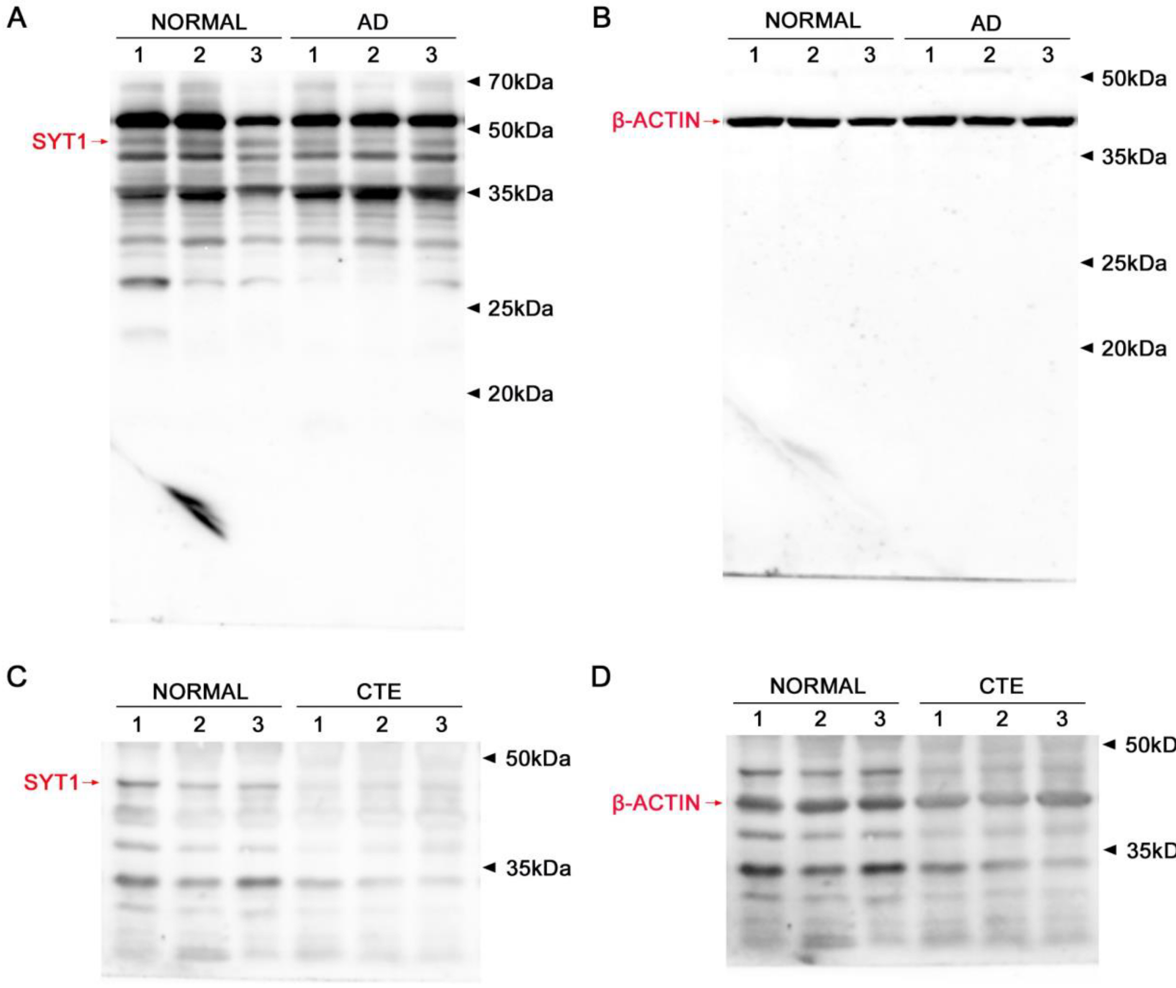

Supplementary Fig. S3. The whole Western blot images of SYT1 and β-ACTIN that are presented in the Fig. 3F.

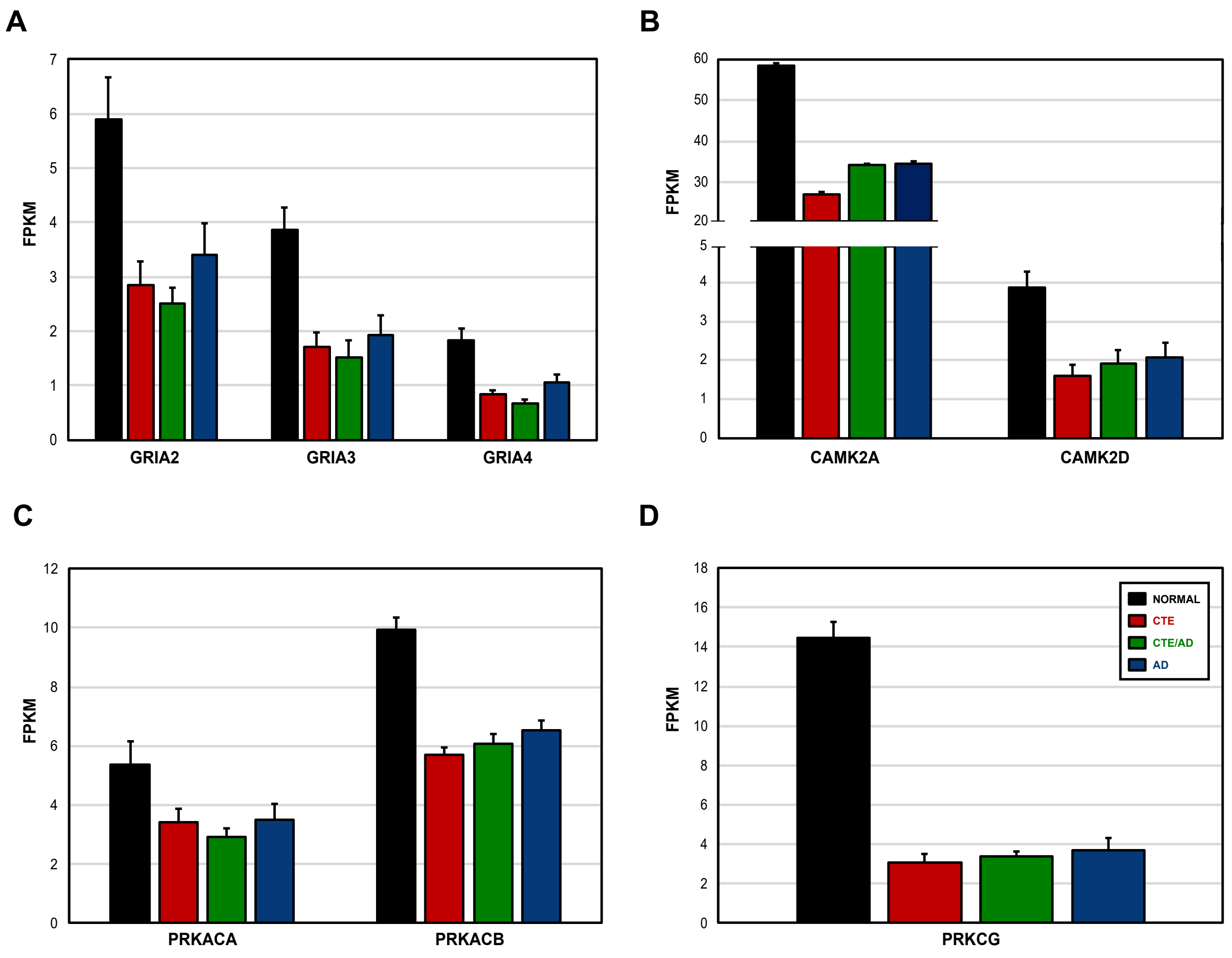

Supplementary Fig. S4. FPKM levels of LTP-related genes in CTE, CTE/AD and AD.
(A) AMPAR gene (GRIA2, GRIA3, and GRIA4) (B) CAMKII genes (CAMK2A, CAMK2D) (C) PKA genes (PRKACA, PRKACB) (D) PKC gene (PRKCG)
